# A Novel Cross-Linked Hemoglobin-Based Oxygen Carrier, YQ23, Extended the Golden Hour for Uncontrolled Hemorrhagic Shock in Rats and Miniature Pigs

**DOI:** 10.3389/fphar.2021.652716

**Published:** 2021-05-12

**Authors:** Lei Kuang, Yu Zhu, Yue Wu, Kunlun Tian, Xiaoyong Peng, Mingying Xue, Xinming Xiang, Billy Lau, Fei Chuen Tzang, Liangming Liu, Tao Li

**Affiliations:** ^1^ State Key Laboratory of Trauma, Burns and Combined Injury, Department of Shock and Transfusion, Daping Hospital, Third Military Medical University (Army Medical University), Chongqing, China; ^2^ New Beta Innovation Limited, Chevalier Commercial Center, Kowloon Bay, Hong Kong, China

**Keywords:** hemoglobin-based oxygen carrier (HBOC), uncontrolled hemorrhagic shock, fluid resuscitation, early treatment, oxygen delivery

## Abstract

**Background:** Hypotensive resuscitation is widely applied for trauma and war injury to reduce bleeding during damage-control resuscitation, but the treatment time window is limited in order to avoid hypoxia-associated organ injury. Whether a novel hemoglobin-based oxygen carrier (HBOC), YQ23 in this study, could protect organ function, and extend the Golden Hour for treatment is unclear.

**Method:** Uncontrolled hemorrhagic shock rats and miniature pigs were infused with 0.5, 2, and 5% YQ23 before bleeding was controlled, while Lactate Ringer’s solution (LR) and fresh whole blood plus LR (WB + LR) were set as controls. During hypotensive resuscitation the mean blood pressure was maintained at 50–60 mmHg for 60 min. Hemodynamics, oxygen delivery and utilization, blood loss, fluid demand, organ function, animal survival as well as side effects were observed. Besides, in order to observe whether YQ23 could extend the Golden Hour, the hypotensive resuscitation duration was extended to 180 min and animal survival was observed.

**Results:** Compared with LR, infusion of YQ23 in the 60 min pre-hospital hypotensive resuscitation significantly reduced blood loss and the fluid demand in both rats and pigs. Besides, YQ23 could effectively stabilize hemodynamics, and increase tissue oxygen consumption, increase the cardiac output, reduce liver and kidney injury, which helped to reduce the early death and improve animal survival. In addition, the hypotensive resuscitation duration could be extended to 180 min using YQ23. Side effects such as vasoconstriction and renal injury were not observed. The beneficial effects of 5% YQ23 are equivalent to similar volume of WB + LR.

**Conclusion:** HBOC, such as YQ23, played vital roles in damage-control resuscitation for emergency care and benefited the uncontrolled hemorrhagic shock in the pre-hospital treatment by increasing oxygen delivery, reducing organ injury. Besides, HBOC could benefit the injured and trauma patients by extending the Golden Hour.

## Introduction

More than half of the early trauma deaths are caused by hemorrhage and 30–40% of deaths occur within 30 minutes after injury (Brohi et al., 2019). With major trauma, hemostatic dressings or tourniquets have limited effects and surgical intervention are needed to stop bleeding, especially with contusion and laceration of parenchymal organs. However, medical measures are limited at the injury site and emergency care for trauma patients, whose bleeding were not controlled, in pre-hospital scenarios has been the focus of trauma researchers and clinicians. In order to reduce bleeding, stabilize the wounded and gain the time for follow-up treatment, damage control resuscitation have been proposed for early fluid resuscitation, including delayed resuscitation and hypotensive resuscitation (Cherkas, 2011; Bogert et al., 2016). Saline, Lactate Ringer’s solution (LR), and hydroxyethyl starch sodium chloride solution (6%, 130/0.4) are conventional fluids for uncontrolled hemorrhagic shock (UHS) resuscitation, but they cannot deliver oxygen and the tissue hypoxia could not be restored. As a result, treatment time window for conventional fluid was small and 90 min is the bottom line for hypotensive resuscitation (Li et al., 2011), otherwise the mean arterial pressure (MAP) cannot be managed well, hemodynamic instability and severe organs injury occurs. Therefore, it is urgent to find new resuscitation fluid that not only reduces bleeding and protects organ functions but also extends the timeframe of pre-hospital treatment.

Blood is an ideal resuscitation fluid for trauma patients, and blood transfusion is believed to increase the blood volume while restoring tissue oxygen supply timely at the same time. However, it is difficult to apply blood transfusion at the injury site or during the evacuation and blood shortage further decreases its availability. Therefore, blood substitutes have become new options in these scenarios. Hemoglobin-based oxygen carriers (HBOCs) are modified hemoglobin with good oxygen-transporting capacity while the side effects of free hemoglobin are reduced. They are easy to store, with higher availability in remote or emergency situations, and may play a huge role in the future (Haldar et al., 2019). Studies have found that HBOC can significantly increase oxygen supply, which is beneficial to trauma and shock. In the previous studies, we used HBOC to treat controlled hemorrhagic shock and septic shock, and found that it could significantly increase tissue oxygen supply and oxygen utilization, restore organ function and increase animal survival (Li et al., 2017; Kuang et al., 2019). We hypothesize that HBOC could benefit UHS in the pre-hospital application.

In this study, a novel bovine-derived tetrameric non-polymerized HBOC, YQ23, was infused in the UHS rats and pigs to observe its performance in the pre-hospital stage treatment for uncontrolled hemorrhagic shock. The aim of this study was to investigate whether the oxygen carrying capacity of YQ23 could benefit the UHS. The volume of blood loss, fluid demand and hemodynamics before bleeding controlled, as well as its effects on tissue oxygen supply and consumption, organ function, and animal survival were observed.

## Materials and Methods

### Ethical Statement

The study was approved by the Laboratory Animal Welfare and Ethics Committee of Third Military Medical University (Army Medical University) according to the guidelines of the ethical use of animals. The investigation conformed to the Guide for the Care and Use of Laboratory Animals (the National Academies Press, eighth edition, 2011), and all rats and pigs were guaranteed to the least suffer.

### HBOC Preparation

The HBOC solution, 5% YQ23 (w/v, [Hb] = 5 g/dl), was provided by New Beta Innovation Limited, Hong Kong. YQ23 is a bovine-derived, stabilized, non-polymerized, cross-linked, cell-free, tetrameric hemoglobin-based oxygen carrier, with undetectable, or low phospholipid, DNA impurities, and protein impurities in the solution and the level of hemoglobin was >99.2%. The molecular weight of YQ23 is 64kDa, and advanced production technology ensures that both amount of large molecular-weight molecules and small molecular-weight molecules (e.g., polymerized hemoglobin or dimeric hemoglobin) are very low, which is helpful for reducing adverse effects, such as renal toxicity. Besides, the stabilized hemoglobin is more resistant to oxidation, and the methemoglobin content in this product is <4.8%. The pH of the product in this study ranged from 7.3 to 7.6, and the p50 was approximately 40 mmHg. The osmolality (at 37°C) was >250 mOsm/kg and the viscosity was 0.9cP. Other information regarding the YQ23 product is shown in patent no. US7,932,356 B1, US 8,048,856 B1, and PCT/US12/46,130. According to our previous study, YQ23 was further diluted into 0.5 and 2% with Lactate Ringer’s solution (LR) before use ([Hb] = 0.5 g/dl and 2 g/dl).

## The Design of the Experiment

The study was performed on both male and female Sprague-Dawley rats (weight: 220–240 g) and miniature pigs (weight: 24–30 kg) obtained from the Animal Center of Army Medical Center. Animals were caged in a specific-pathogen-free caring-room at the temperature of 24 ± 1°C and light cycle (6 AM–6 PM) and were fed standard pellet diets *ad libitum*.

**FIGURE 1:**
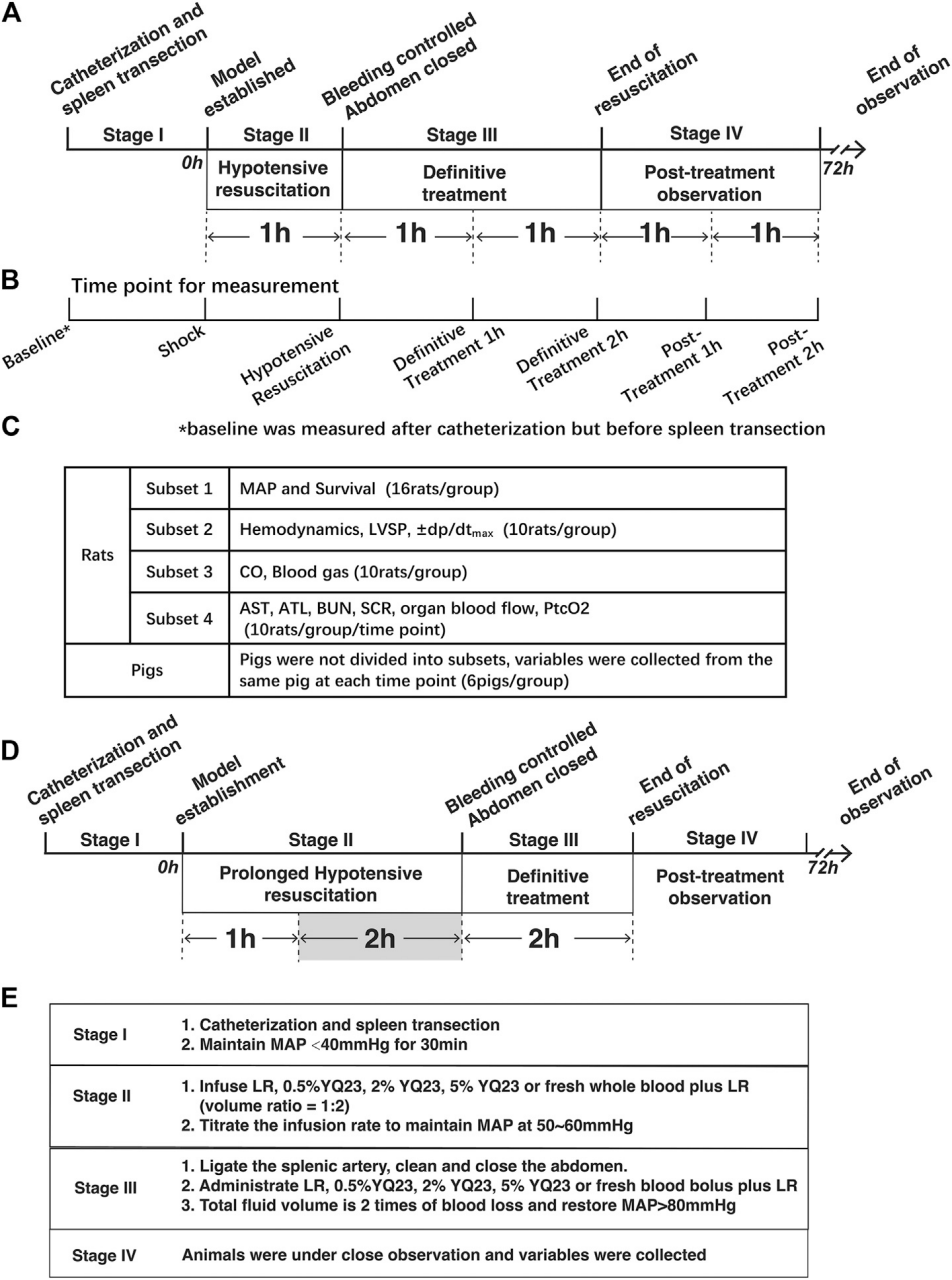
**Schematics of the study design. (A)**, Staged Treatment in Experiment Part 1, rats and pigs undergo staged treatment. In Stage I, pigs and rats were catheterized and the spleen transection was performed to replicate the uncontrolled hemorrhagic shock model. In Stage II, the hypotensive resuscitation was carried out for 60 min, during which YQ23, LR, and whole blood plus LR were infused to restore MAP to 50–60 mmHg. In Stage III, bleeding was controlled and definitive treatment was carried out, the above fluids were further infused for the next 120 min and the total fluid volume is two times of blood loss. In Stage IV, animals were under close observation when treatment was ended. Variables were collected during and after treatment. Data of rats were collected form 4 subsets experiments. **(B)**, Time point for variable collection, variables were collected at the 7 indicated time point; **(C)**, Design of rat experiment subsets, rats were divided into 4 subsets for the indicated variable collection at each time point; **(D)**, Staged-treatment of Experiment Part 2, the hypotensive resuscitation duration was extended to 180 min and the definitive treatment also lasted for 120 min to observe whether YQ23 could gain time for subsequent treatment. **(E)**, staged treatment workflow, a brief list of treatments in the corresponding stage was shown. LR, Lactate Ringer’s solution; MAP, mean arterial pressure.

The study consists of two parts: The Experiment Part 1 was designed to investigate the protective effects of YQ23 on UHS in a routine resuscitation strategy. Both rats and pigs were used to replicate model, and UHS animals underwent staged treatment, including model establishment, pre-hospital treatment (hypotensive resuscitation), definitive treatment (bleeding control and further fluid therapy) and post-resuscitation stage. Variables were collected during the staged treatment. When the therapeutic effects of YQ23 was confirmed, the Experiment Part 2 on murine model was designed to investigate whether YQ23 could extend the Golden Hour for trauma treatment and the hypotensive resuscitation was increased to 180 min. The design of experiment is listed below and in Figure 1.

### Experiment Part 1

#### Staged Treatment for Rats

In stage I, animals were anaesthetized and UHS models were built. Rats were anesthetized with sodium pentobarbital (30 mg/kg, intraperitoneal) until they were unresponsive to needle stimulus. Then, the right femoral artery and vein were catheterized with polyethylene tubule (PE9050, inner diameter = 0.5 mm, SDR scientific) for mean arterial pressure (MAP) monitoring and fluid administration, respectively. The UHS model was induced by transection of the splenic parenchyma as previously described (Li et al., 2012). Briefly, a cross-transection in the rat splenic parenchyma between the two major branches of the splenic artery was made and one of the major branches of the splenic artery was also transected. Blood was allowed to flow into the abdominal cavity freely, and the abdomen was left open (the incision was aligned but not sutured) and covered with warm wet gauze pad. When the MAP decreased to below 40 mmHg and lasted for 30 min, the model was considered built.

In stage II (pre-hospital stage), the 60 min-hypotensive resuscitation was carried out. Model animals were randomly assigned to LR and 0.5% YQ23, 2% YQ23, 5% YQ23, and whole blood plus LR (WB + LR) group for resuscitation. The fluid was infused with infusion syringe pump (Perfusor^®^ compact S). In rat experiment, the initial infusion rate was 10 ml/h for LR, 4 ml/h for 0.5% YQ23, 2 ml/h for 2%YQ23, 1.5 ml/h for 5% YQ23. Fresh whole blood (WB) was given along with LR, and rats received a bolus of 500 μL fresh blood from the donor rats followed by 1 ml LR (LR: WB = 2:1) each time. The infusion rate and the interval for blood transfusion was titrated to maintain MAP at 50–60 mmHg.

In stage III (hospital stage), the 120 min definitive treatment was performed, in which the splenic artery was ligated, the abdominal cavity was cleaned and closed while the volume of blood loss was recorded (by weighing the blood and clot in the abdominal cavity). Meanwhile, LR, 0.5% YQ23, 2% YQ23, 5% YQ23 and WB + LR were further administrated for restoring hemodynamics and expanding volume. The total infusion volume (in stage II and III) was two times of blood loss and MAP was restored over 80 mmHg.

In stage IV, animals were under close observation for 120 min and variables were further collected. Strict sterile manipulation was employed and animals were subjected to the least suffer during the whole experiment. The survived animals after experiments were sacrificed by decapitation after anesthesia.

#### Staged Treatment for Pigs

Because the size of rat is small and there is a difference between rats and human beings in the physiological indices, we further used large animals, miniature pigs, whose physiology is closer to human beings, to repeated the staged treatment.

In stage I, pigs were anesthetized with ketamine (10 mg/kg, intramuscular) and diazepam (0.4 mg/kg, intramuscular) followed by sodium pentobarbital (30–50 mg/kg, intravenous) and the right femoral artery and vein were catheterized with polyethylene tubule (PE240, inner diameter = 0.66in, BD Intramedic ™). The UHS model was also induced by transection of the splenic parenchyma as previously described. When the MAP decreased to below 40 mmHg and lasted for 30 min, the model was established.

In stage II, in the 60 min hypotensive resuscitation, the initial infusion rate was 90 ml/h for LR, 30 ml/h for 0.5%, 20 ml/h for 2% YQ23, 5 ml/h for 5% YQ23. Besides, in WB group, they received 2.5 ml fresh blood from the donor pig followed by 5 ml LR (LR:WB = 2:1) every time. The MAP was maintained at 50–60 mmHg by adjusting the infusion rate.

In stage III, 120 min definitive treatment was carried out and the abdomen cavity was cleaned and closed, blood loss was recorded and fluid infusion was continued. The total infusion fluid volume was also two times of the blood loss. In stage IV, the pigs were also closely observed for 120 min. After 72 h observation, survived pigs were sacrificed by infusing solutions of potassium chloride under anesthesia.

### Experiment Part 2: Prolonged Hypotensive Resuscitation

After the therapeutic effect of YQ23 was confirmed, the Experiment Part 2 was carried out and was aimed to investigate whether YQ23 could extend the hypotensive resuscitation duration. In the Experiment Part 2, only murine model was employed, and the hypotensive resuscitation duration was extended to 180 min in stage II (Figure 1). Other treatments were remained same as experiment part 1.

## Variable Collection

Because the small size of rats, some parameters could not be obtained from the same rats. The data were collected from 4 subsets of rats in Experiment Part 1:

Subset 1: *N* = 16/group, MAP, blood loss, fluid demand and survival were observed.

Subset 2: *N* = 10/group. Hemodynamics including LVSP, ±dp/dt_max_ were observed.

Subset 3: *N* = 10/group. CO, heart rate and blood gas were observed.

Subset 4: *N* = 10/group/time point. Organ blood flow, biochemical items, and PtcO_2_ were observed.

Pigs were not divided into subsets, and *N* = 6/group was used, data were collected together.

### MAP and Survival Time

For both rats and pigs, mean arterial pressure was measured with polygraph physiological recorder (SP844; Power Laboratory, AD Instruments) through the right femoral artery catheter. When resuscitation was ended, animals were placed back to cages and the survival time was recorded.

### Hemodynamics

For both rats and pigs, LVSP was assessed by measuring the intraventricular pressure with a PE catheter that had been inserted into the left ventricle via the right carotid artery. This catheter was connected to a pressure probe (MLT844, PowerLab, AD Instruments) and a polygraph physiological recorder (SP844; PowerLab, AD Instruments). The ± dp/dt_max_ were obtained using LabChart software.

### Cardiac Output

In rats, the cardiac output (CO) was measured with thermodilution techniques as described previously (Liu et al., 2016). A thermodilution probe was inserted into the aorta ascendens of the rat through the right carotid artery, and 0.3 ml ice-bathed saline was injected through the right external jugular vein catheter. The CO was determined using a CO analyzer (Power Laboratory, AD Instruments, Australia). However, some rats in the LR group died before the observation ended and resulted in reduced sample size. The accurate sample size was illustrated in the corresponding figures.

For pigs, a Swan-Ganz catheter (SP5105H Thermal Dilution catheter, CritiCath, Becton Dickinson) was inserted into the right atrium from the right carotid vein and 5 ml ice-bathed saline was injected to measure CO. Heart rate (HR) were recorded by a polygraph physiological recorder (SP844; Power Laboratory, AD Instruments). Cardiac index (CI) and stroke index (SI) were calculated with the following equations: *CI* = CO/S, *S* = 9.1 × *W* ^2/3^, *SI* = CI/HR, in which *S* represents the body surface, and *W* represents the bodyweight (Li et al., 2017).

### Blood Gas, DO_2_, VO_2_

Data were collected from the same rats and pigs for cardiac output measurement. Arterial and venous blood gas (PaO_2_, SaO_2_, SvO_2_, Lac, Hb) were determined with the blood gas analyzer (ABL 800 FLEX; Radiometer, Copenhagen, Denmark). To avoid additional blood loss in rats and pigs, an equal volume of blood obtained from donor animals was infused back right after sampling. Oxygen delivery (DO_2_) and utilization (VO_2_) were calculated with the following equations: DO_2_ = CI × 13.4 × [Hb] × SaO_2_; VO_2_ = CI × 13.4 × [Hb] × (SaO_2_–SvO_2_).

### Organ Blood Flow

For rats, the blood flow of the liver, kidney, and brain was measured by a laser Doppler system (Periflux System 5000; Perimed, Stockholm, Sweden). The abdomen and cranial cavity were opened and the blood flow probes were place on the surface of vital organs. Because it is an invasive measurement which causes more injury and might influence the survival observation of pigs, blood flow of brain, liver and kidney was not investigated in pigs.

### Renal Function, Liver Injury Indices and PtcO_2_

The blood was collected to determine blood urea nitrogen (BUN), serum creatinine (SCR), aspartate aminotransferase (AST), and alanine aminotransferase (ALT) with Biochemical Analyzer (DX800; Beckman Coulter, Fullerton, CA). The transcutaneous partial pressure of oxygen (PtcO_2_) was determined by a laser Doppler system (Periflux System 5000; Perimed, Stockholm, Sweden) and the probes were place on the depilated skin (Kuang et al., 2019).

## Statistical Analysis

Parametric data (MAP, blood gas, DO_2_, VO_2_, PtcO_2_, cardiac function, AST, ALT, BUN, and SCR) were presented as the mean ± standard deviation of n observations. The statistical differences among groups were analyzed by two-factor variance analysis, followed by the post-hoc Tukey test (SPSS v15.0; SPSS Inc., Chicago, IL) for multiple comparisons between groups. Survival was analyzed by Kaplan-Meier survival analysis and the log-rank test, survival time was presented as median with interquartile range. *p* < 0.05 was considered significant.

## Results

### YQ23 Reduced the Volume of Bleeding and Fluid Demand in UHS Rats and Pigs

**FIGURE 2:**
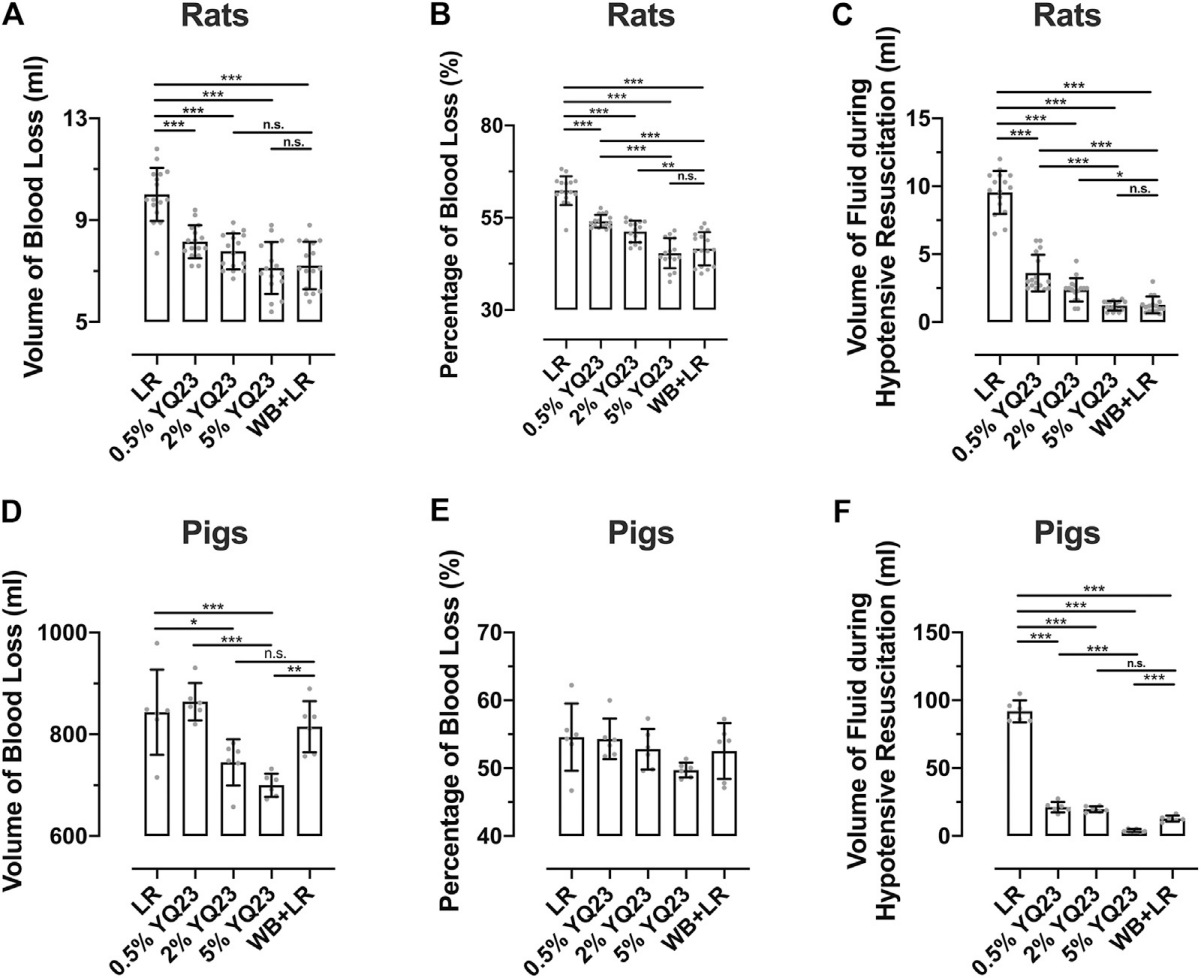
**YQ23 decreased the blood loss and fluid demand during hypotensive resuscitation following uncontrolled hemorrhagic shock.** (*n* = 16/group in rat experiment, *n* = 6/group in pig experiment). **(A)**, the volume of blood loss in rats; **(B)**, percentage of blood loss in rats; **(C)**, the volume of fluid required during hypotensive resuscitation in rats; **(D)**, the volume of blood loss in pigs; **(E)**, percentage of blood loss in pigs; **(F)**, the volume of fluid required during hypotensive resuscitation in pigs. LR, Lactate Ringer’s solution; WB, whole blood. *p < 0.05, **p < 0.01, ***p < 0.001, n. s. not significant, ^#^ n of LR group at the corresponding time point (sample size was decreased because of animal death).

Fluid resuscitation and transfusion are of the most valuable means to save lives under uncontrolled hemorrhagic shock (UHS). Large-volume infusion on the battlefield is difficult to achieve and increases bleeding (Stern et al., 1995). To clarify the effects of different fluids on bleeding and fluid demand, we recorded the volume of blood loss and the corresponding fluid demand during hypotensive resuscitation. The results showed that YQ23 and WB + LR could significantly reduce blood loss in rats compared with LR, the blood loss proportion was 45.38 ± 4.08%, 46.58 ± 4.50% and 62.33 ± 3.93% in 5% YQ23, WB + LR and LR group, respectively, (Figures 2A,B). During the hypotensive resuscitation, more LR was required to maintain the target blood pressure, the volume of infused LR was 9.54 ± 1.58 ml. In YQ23 groups, fluid demand was significantly decreased, and the volume of 5% YQ23 was only about 1/8 of the amount in LR group, which was equivalent to WB + LR (Figure 2C). Besides, the total fluid volume for resuscitation was two times of blood loss and they were 18.3 ± 4.10 ml for LR, 16.3 ± 1.29 ml for 0.5%YQ23, 15.55 ± 1.41 ml for 2%YQ23, and there was no significant difference between 5% YQ23 (14.25 ± 2.04 ml) and WB + LR (14.43 ± 1.86 ml). These results suggested that the effects of 5% YQ23 were equivalent to WB + LR.

In the UHS pigs, the trend in blood loss and fluid demand was similar to rats. The blood loss was significantly reduced by 5% YQ23 (Figures 2D,E) compared with LR and WB + LR, the blood loss proportion was 49.74 ± 1.11%, 52.53 ± 4.11%, and 54.57 ± 4.96% in 5% YQ23, WB + LR and LR group, respectively. Besides, the LR group demonstrated the highest fluid demand during hypotensive resuscitation, and the 5% YQ23 group demonstrated the lowest fluid demand (Figure 2F).

### YQ23 Stabilized the Hemodynamics of UHS Animals

**FIGURE 3:**
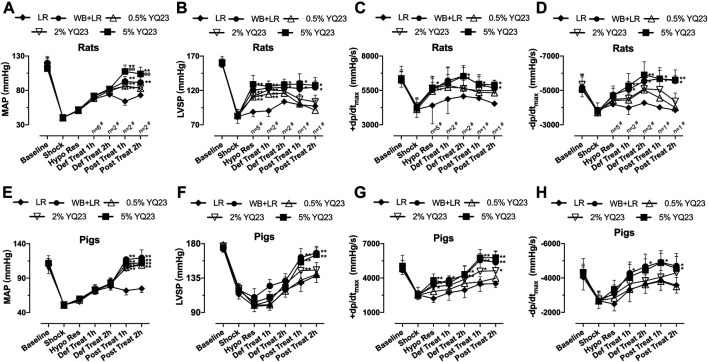
**YQ23 restored hemodynamics following uncontrolled hemorrhagic shock in rats and pigs. (A)**, MAP in rats (*n* = 16/group unless otherwise annotated); **(B)**, LVSP in rats (*n* = 10/group unless otherwise annotated); **(C,D)**, ±dp/dt_max_ in rats (*n* = 10/group unless otherwise annotated); **(E)**, MAP in pigs (*n* = 6/group); **(F)**, LVSP in pigs (*n* = 6/group); **(G,H)**, ±dp/dt_max_ in pigs (*n* = 6/group). MAP, mean arterial pressure; LVSP, left ventricular systolic pressure; ±dp/dt_max_, maximal rate of the increase or decrease of left ventricular pressure; LR, Lactate Ringer’s solution; WB, whole blood. *p < 0.05 v. s. LR at the same time point, **p < 0.01 v. s. LR at the same time point, ^@^ *p* < 0.05 v. s. WB + LR at the same time point, ^@@^ *p* < 0.01 v. s. WB + LR at the same time point.

We observed the effects of different fluids on restoring hemodynamic in rats, which is important for reducing early casualties following UHS. During hypotensive resuscitation, YQ23 and LR could maintain MAP at 50–60 mmHg. After definitive treatment, MAP could not be maintained in LR group, and trended to decrease, and finally maintained at 60–76 mmHg, indicating that crystal fluid alone cannot effectively maintain blood pressure. YQ23 maintained MAP at a higher level compared with LR. 5% YQ23 could restore MAP to 92–126 mmHg, which was close to the baseline and significantly higher than WB + LR (Figure 3A). Further observations were made on the changes of LVSP and ±dp/dt_max_ in rats at different resuscitation stages. These indices can reflect the heart systolic, diastolic, and overall circulatory stability. LR demonstrated limited ability to improve LVSP and ±dp/dt_max_: after hypotensive resuscitation, the above parameters remained at the shock level, and there was even a downward tendency. YQ23 significantly increased LVSP and ±dp/dt_max_ during the hypotensive resuscitation and definitive treatment, the LVSP and ±dp/dt_max_ were maintained after treatment (Figures 3B–D). There was no significant difference between 5% YQ23 and WB + LR.

In the UHS pig models, results were similar to that of rats. LR slightly restored MAP, LVSP, and ±dp/dt_max_. YQ23 and WB + LR demonstrated significantly better effects. In phase IV, the MAP of pigs was maintained at 90–126 mmHg in YQ23 groups, at 106–140 mmHg in WB + LR group, which were significantly higher than LR group (64–80 mmHg) (Figure 3E). Also, both LVSP and ±dp/dt_max_ in YQ23 and WB + LR group were restored to near normal level, which were significantly higher than those of the LR group (Figures 3F–H). There was no significant difference between 5%Y23 and WB + LR. The above results indicated that YQ23 could maintain and stabilize hemodynamics, and the effects of 5% YQ23 were equivalent to WB + LR.

### YQ23 Improved Systemic Oxygen Supply, Consumption and Blood Flow in UHS Animals

Whether the oxygen-carrying function could benefit UHS is another key point that deserves attention. We observed the blood gas and found that after infusion of YQ23, the PaO_2,_ and SaO_2_ were restored rapidly, Lac were reduced, and pH gradually recovered to 7.30–7.33. Besides, WB + LR could rapidly increase rat PaO_2_, SaO_2_, correct acidosis, and there was no significant difference between 5%YQ23 and WB + LR. On the other hand, PaO_2_ and SaO_2_ in LR group remained at the shock level and Lac even had an increasing tendency (Table 1).

**TABLE 1:** YQ23 restored blood gas index in UHS animals.

	Baseline	Shock	Hypotensive resuscitation	Definitive treatment 1 h	Definitive treatment 2 h	Post treatment 1 h	Post treatment 2 h
pH							
LR	7.38 ± 0.06	7.38 ± 0.03	7.22 ± 0.01 (*n* = 5)	7.21 ± 0.04 (*n* = 2)	7.26 ± 0.09 (*n* = 2)	7.35 (*n* = 1)	7.37 (*n* = 1)
0.50% YQ23	7.39 ± 0.06	7.39 ± 0.04	7.29 ± 0.06	7.24 ± 0.08	7.26 ± 0.04	7.30 ± 0.02^@^	7.28 ± 0.05^@@^
2% YQ23	7.38 ± 0.05	7.40 ± 0.05	7.29 ± 0.07	7.30 ± 0.09**	7.27 ± 0.07	7.32 ± 0.01	7.33 ± 0.08
5% YQ23	7.38 ± 0.05	7.39 ± 0.03	7.29 ± 0.04**	7.29 ± 0.09*	7.23 ± 0.07	7.33 ± 0.03	7.32 ± 0.04
WB + LR	7.38 ± 0.05	7.35 ± 0.04	7.30 ± 0.03**	7.23 ± 0.09**	7.31 ± 0.08	7.37 ± 0.05	7.38 ± 0.04
Hb (g/dl)							
LR	11.87 ± 0.40	9.58 ± 0.18	9.06 ± 0.24 (*n* = 5)	8.35 ± 0.49 (*n* = 2)	7.55 ± 0.49 (*n* = 2)	7.7 (*n* = 1)	6.7 (*n* = 1)
0.50% YQ23	11.76 ± 0.55	9.60 ± 0.31	9.86 ± 0.68* ^,^ ^@@^	9.55 ± 0.91^@@^	9.03 ± 0.34^@@^	9.02 ± 0.17^@@^	8.64 ± 0.25^@@^
2% YQ23	11.54 ± 0.62	9.42 ± 0.22	9.87 ± 0.68* ^,^ ^@@^	10.05 ± 0.40^@@^	9.40 ± 0.28^@@^	9.43 ± 0.24^@@^	9.03 ± 0.29^@@^
5% YQ23	11.44 ± 0.76	9.89 ± 0.80	10.04 ± 0.43** ^,^ ^@@^	10.08 ± 0.39^@@^	9.53 ± 0.21^@@^	9.50 ± 0.19^@@^	9.02 ± 0.27^@@^
WB + LR	11.78 ± 0.67	10.05 ± 0.69	11.25 ± 0.23**	10.85 ± 0.45	10.88 ± 0.4	11.02 ± 0.4	10.83 ± 0.29
PaO_2_ (mmHg)							
LR	96.66 ± 7.07	97.96 ± 4.22	71.26 ± 2.51 (*n* = 5)	71.1 ± 6.36 (*n* = 2)	73.8 ± 5.09 (*n* = 2)	70.5 (n = 1)	68.6 (n = 1)
0.50% YQ23	97.83 ± 7.78	98.51 ± 6.7	78.62 ± 8.27	85.17 ± 14.05	80.87 ± 5.33	70.82 ± 6.3	74.49 ± 17.29
2% YQ23	96.52 ± 7.74	99.27 ± 9.59	92.48 ± 14.44**	94.31 ± 18.44	92.29 ± 14.14	94.80 ± 5.61	98.76 ± 15.94
5% YQ23	99.65 ± 10.98	98.06 ± 7.90	91.11 ± 9.76**	94.23 ± 10.92	94.44 ± 6.58	95.38 ± 7.39	98.56 ± 14.11
WB + LR	100.32 ± 10.53	99.25 ± 8.92	87.61 ± 8.01**	98.22 ± 6.64	92.36 ± 12.05	93.61 ± 8.15	85.95 ± 23.78
SaO_2_ (%)							
LR	95.34 ± 1.36	97.50 ± 1.41	89.84 ± 0.83 (*n* = 5)	83.75 ± 1.63 (*n* = 2)	80.25 ± 7.57 (*n* = 2)	74.1 (*n* = 1)	70.6 (*n* = 1)
0.50% YQ23	96.66 ± 1.27	95.48 ± 1.60	94.25 ± 0.91**	93.33 ± 3.16*	93.10 ± 2.38^@@^	93.22 ± 1.38^@@^	91.18 ± 1.67
2% YQ23	95.69 ± 2.01	94.97 ± 1.83	94.83 ± 1.13**	94.29 ± 1.55	95.06 ± 1.41	94.22 ± 0.94^@^	92.11 ± 2.51
5% YQ23	96.00 ± 1.35	95.18 ± 1.64	95.57 ± 1.28**	95.43 ± 1.86	94.73 ± 1.35^@^	94.65 ± 1.82	93.62 ± 2.44
WB + LR	96.25 ± 2.22	96.24 ± 2.97	96.34 ± 4.85*	95.52 ± 2.29*	96.85 ± 1.35	96.38 ± 1.81	94.27 ± 3.32
Lac (mmol/L)							
LR	2.59 ± 0.20	7.90 ± 0.19	6.24 ± 0.97 (*n* = 5)	6.35 ± 1.34 (*n* = 2)	6.45 ± 0.21 (*n* = 2)	8.1 (*n* = 1)	7.2 (*n* = 1)
0.50% YQ23	2.57 ± 0.28	7.84 ± 0.35	5.98 ± 0.73	6.04 ± 0.87^@@^	5.28 ± 0.52* ^,^ ^@^	5.04 ± 0.57	4.82 ± 0.49
2% YQ23	2.71 ± 0.26	7.80 ± 0.28	5.34 ± 0.82^@^	5.31 ± 0.81^@@^	5.26 ± 0.50* ^,^ ^@^	5.21 ± 0.54^@@^	4.92 ± 0.41
5% YQ23	2.82 ± 0.23	7.78 ± 0.30	6.89 ± 0.43	5.60 ± 0.60^@@^	5.86 ± 0.52^@@^	5.80 ± 0.36^@@^	4.71 ± 0.74
WB + LR	2.47 ± 0.31	7.71 ± 0.52	6.70 ± 0.94	3.81 ± 0.75	4.42 ± 0.66**	4.37 ± 0.39	4.38 ± 0.61

*N* = 10, unless otherwise annotated (sample size was reduced because of animal death); LR, Lactate Ringer’s solution; WB, whole blood.

* *p* < 0.05 v. s. LR at the same time point.

** *p* < 0.01 v. s. LR at the same time point.

@ *p* < 0.05 v. s. WB + LR at the same time point.

@@ *p* < 0.01 v. s. WB + LR at the same time point.

**FIGURE 4:**
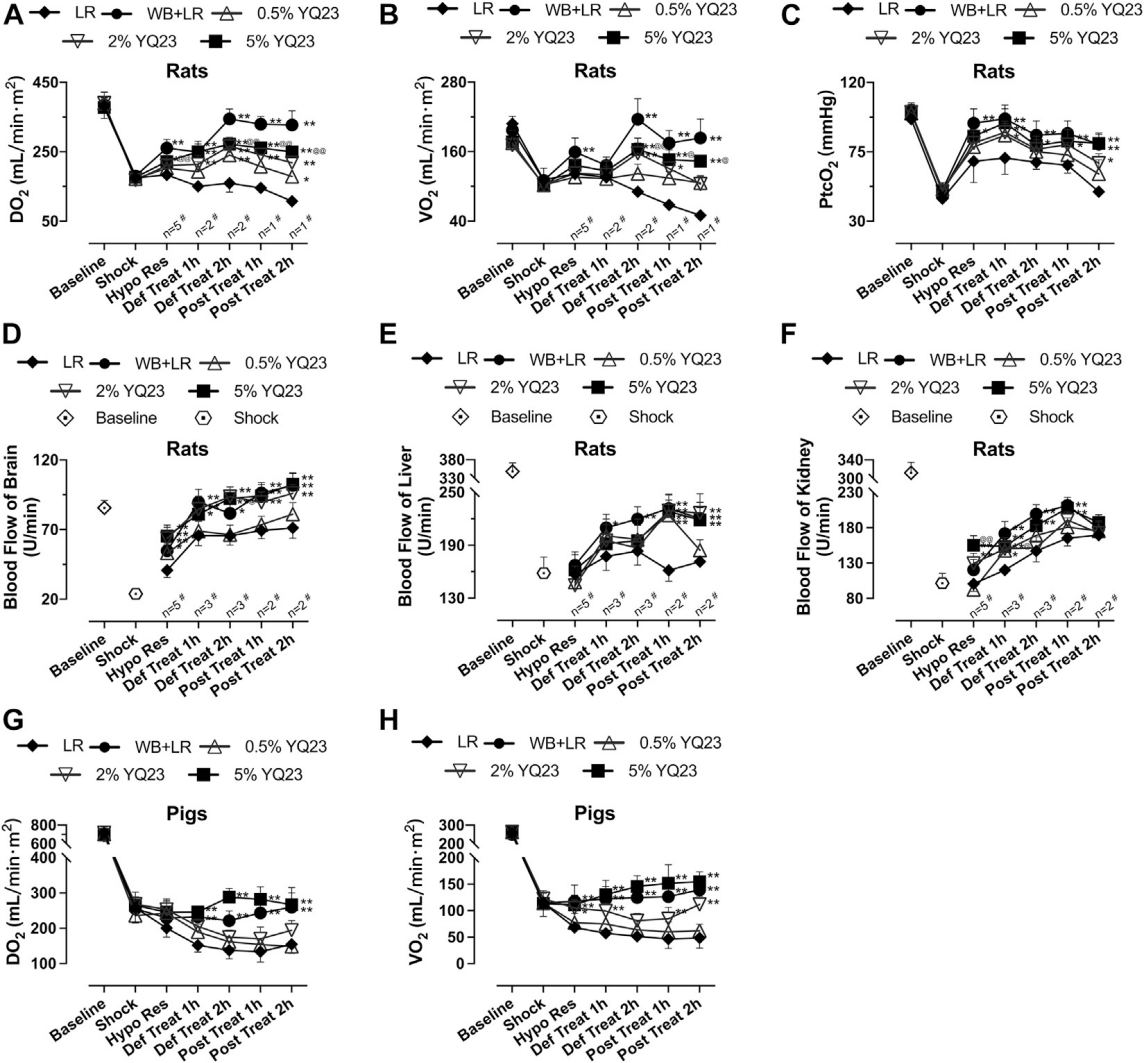
**YQ23 restored oxygen delivery, oxygen utilization, and organ blood perfusion following uncontrolled hemorrhagic shock in rats and pigs. (A)**, DO_2_ in rats; **(B)**, VO_2_ in rats; **(C)**, PtcO_2_ in rats (*n* = 2 for LR group); **(D)**, the blood flow of the brain in rats; **(E)**, the blood flow of the liver in rats; (F), the blood flow of the kidney in rats; **(G)**, DO_2_ in pigs; **(H)**, VO_2_ in pigs (*n* = 10/group in rats experiment unless otherwise annotated, n = 6/group in pig experiment). DO_2_, oxygen delivery; VO_2_, oxygen consumption; PtcO_2_, the transcutaneous partial pressure of oxygen LR, Lactate Ringer’s solution; WB, whole blood. *p < 0.05 v. s. LR at the same time point, **p < 0.01 v. s. LR at the same time point, ^@^ *p* < 0.05 v. s. WB + LR at the same time point, ^@@^ *p* < 0.01 v. s. WB + LR at the same time point, ^#^ n of LR group at the corresponding time point (sample size was decreased because of animal death).

DO_2_ reflects the ability of blood to transport oxygen and is a sensitive predictor of mortality; VO_2_ reflects the tissue’s ability to utilize oxygen and is an indicator of the systemic oxygen metabolism state (Rivers et al., 2015). In this study, YQ23 could increase DO_2_ and VO_2_ in rats, and the DO_2_ in the 2% YQ23 and 5% YQ23 groups was significantly higher than that in LR group. After treatment, DO_2_ was maintained in YQ23 and WB + LR group but decreased in LR group (Figure 4A). Besides, the VO_2_ gradually increased in YQ23 and WB + LR group during resuscitation, suggesting the recovery of aerobic metabolism (Figure 4B). It was found that the PtcO_2_ in YQ23 and WB + LR group were significantly higher than the LR group during hypotensive resuscitation, suggesting the recovery of tissue oxygen supply. Besides, the highest PtcO_2_ level in LR group was only about 68.45% of the baseline level, and in 5% YQ23 and WB + LR they were 94.05 and 96.80% (Figure 4C).

In addition, we observed the blood flow of rat liver, kidney, and brain. 2% YQ23, 5% YQ23 and WB + LR could increase blood flow to higher levels in a shorter time after infusion, compared with LR (Figures 4D–F). There was no significant difference between 5%YQ23 and WB + LR. The above results show that YQ23 helps to reduce hypoxia in tissues and organs and restore systemic oxygen utilization.

In pig experiment, LR failed to restore DO_2_ and VO_2_. YQ23 significantly increased DO_2_ and VO_2_, which were about 1.85 times and 2.54 times of that in the LR group. The effects of 5% YQ23 were equivalent to WB + LR (Figures 4G,H).

### YQ23 Protected the Heart, Liver, Kidney Function

**FIGURE 5:**
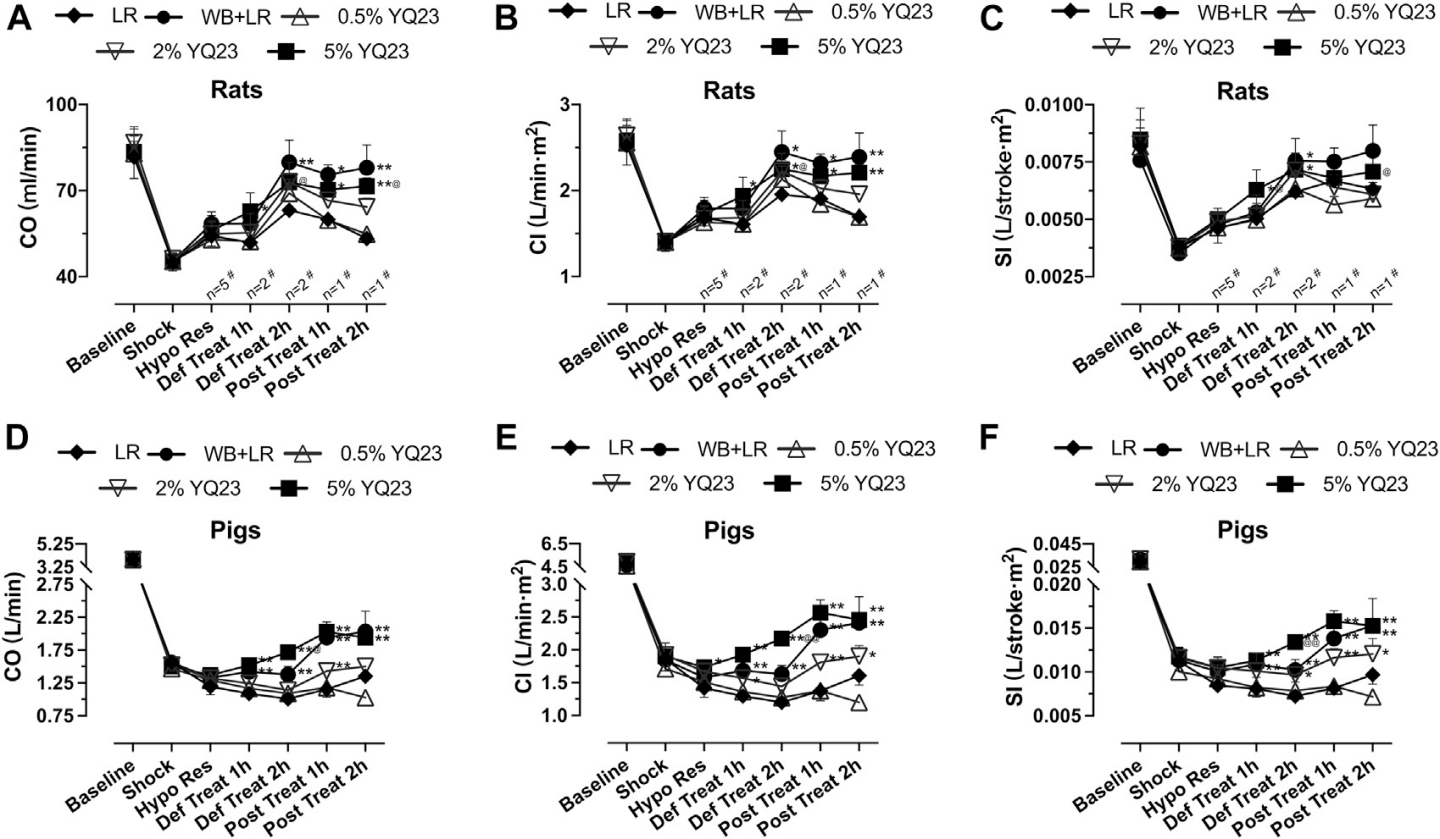
**YQ23 improved cardiac function following uncontrolled hemorrhagic shock in rats and pigs. (A)**, CO in rats; **(B)**, CI in rats; **(C)**, SI in rats; **(D)**, CO in pigs; **(E)**, CI in pigs; **(F)**, SI in pigs (*n* = 10/group in rats experiment unless otherwise annotated, *n* = 6/group in pig experiment). CO, cardiac output CI, cardiac index; SI, stroke index. LR, Lactate Ringer’s solution; WB, whole blood. *p < 0.05 v. s. LR at the same time point, **p < 0.01 v. s. LR at the same time point, ^@^ *p* < 0.05 v. s. WB + LR at the same time point, ^@@^ *p* < 0.01 v. s. WB + LR at the same time point, ^#^ n of LR group at the corresponding time point (sample size was decreased because of animal death).

Cardiac output was used to evaluate heart function. The results showed that CO, CI, and SI in the 2, 5% YQ23 group were increased and maintained at a significantly higher level, compared with LR group. The average CO in LR group was only about 75.6% of the baseline, while in 2% YQ23 and 5% YQ23 group, they were 87.9 and 87.2% of the baseline. The effect of WB + LR was also satisfying, CO could be restored to 95.6% of the baseline (Figures 5A–C).

Similar results were obtained in the UHS pig experiment. The CO, CI, and SI in the YQ23 and WB + LR groups were significantly higher than the LR group, and there was no significant difference between 5%YQ23 and WB + LR (Figures 5D–F). These results show that YQ23 can restore and stabilize cardiac function.

**FIGURE 6:**
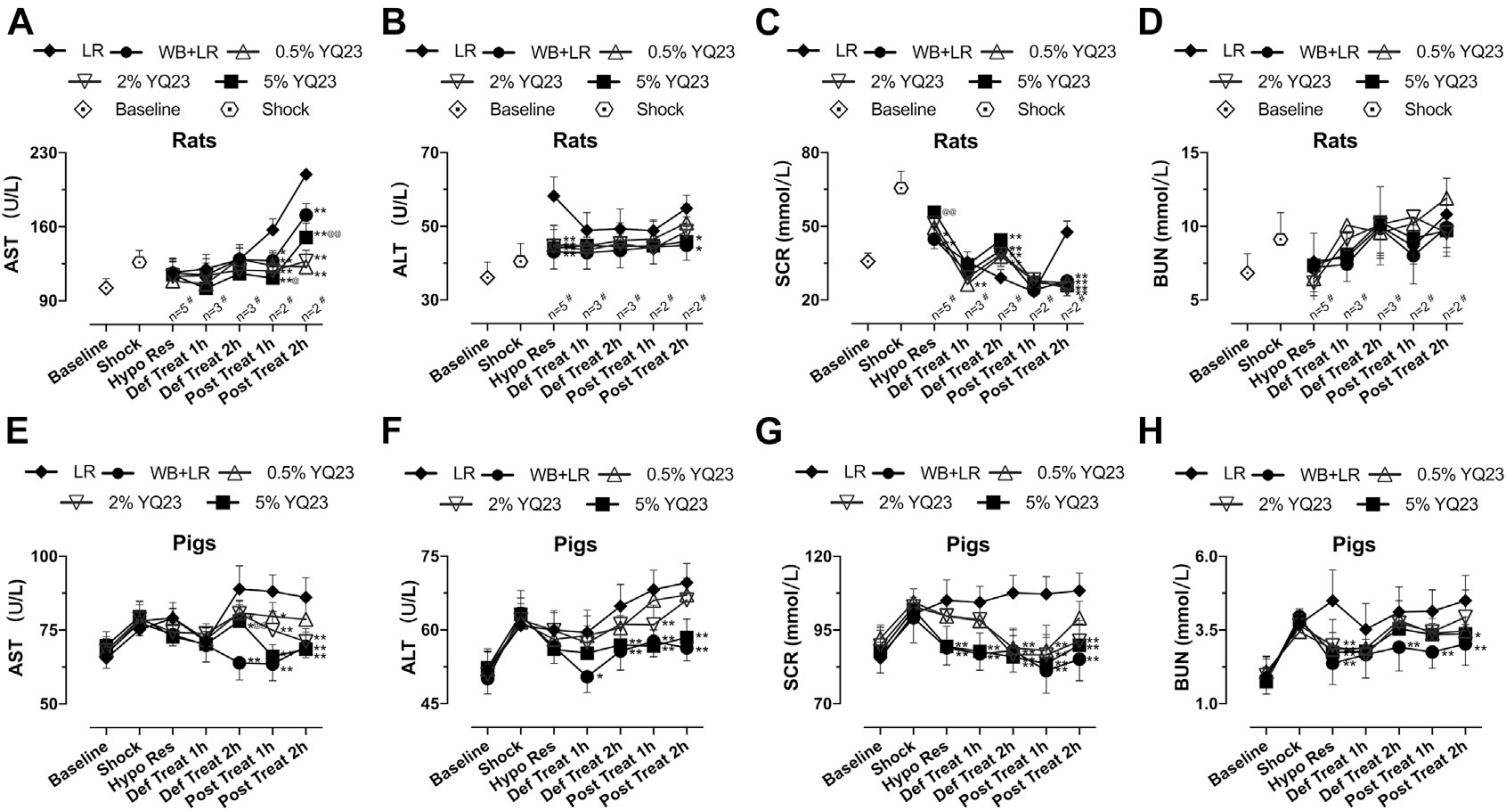
**YQ23 reduced liver and kidney injury following uncontrolled hemorrhagic shock in rats and pigs. (A)**, AST in rats; **(B)**, ALT in rats; **(C)**, SCR in rats; **(D)**, BUN in rats; **(E)**, AST in pigs; **(F)**, ALT in pigs; **(G)**, SCR in pigs; **(H)**, BUN in pigs (*n* = 10/group in rats experiment unless otherwise annotated, *n* = 6/group in pig experiment). AST, aspartate aminotransferase; ALT, alanine aminotransferase; BUN, blood urea nitrogen; SCR, serum creatinine; LR, Lactate Ringer’s solution; WB, whole blood. *p < 0.05 v. s. LR at the same time point, **p < 0.01 v. s. LR at the same time point, ^@^ *p* < 0.05 v. s. WB + LR at the same time point, ^@@^ *p* < 0.01 v. s. WB + LR at the same time point, ^#^ n of LR group at the corresponding time point (sample size was decreased because of animal death).

In order to observe whether YQ23 prevents organ damage, we tested the biochemical items indicating liver and kidney injury. For rats, AST and ALT in WB + LR and YQ23 groups were slightly decreased or maintained at the shock level after treatment, and were significantly lower than LR group, which trended to increase (Figures 6A,B). Besides, increased renal blood flow helped to reduce blood creatinine, the effects of YQ23 and WB + LR were significantly better than LR in the post-resuscitation period, and SCR could be restored near to baseline (Figures 6C,D). There was no significant difference between 5% YQ23 and WB + LR in ALT, AST, and BUN. In experiments on pigs, results were similar (Figures 6E–H).

## YQ23 Reduced Early Death and Increased Survival in UHS Animals

**FIGURE 7:**
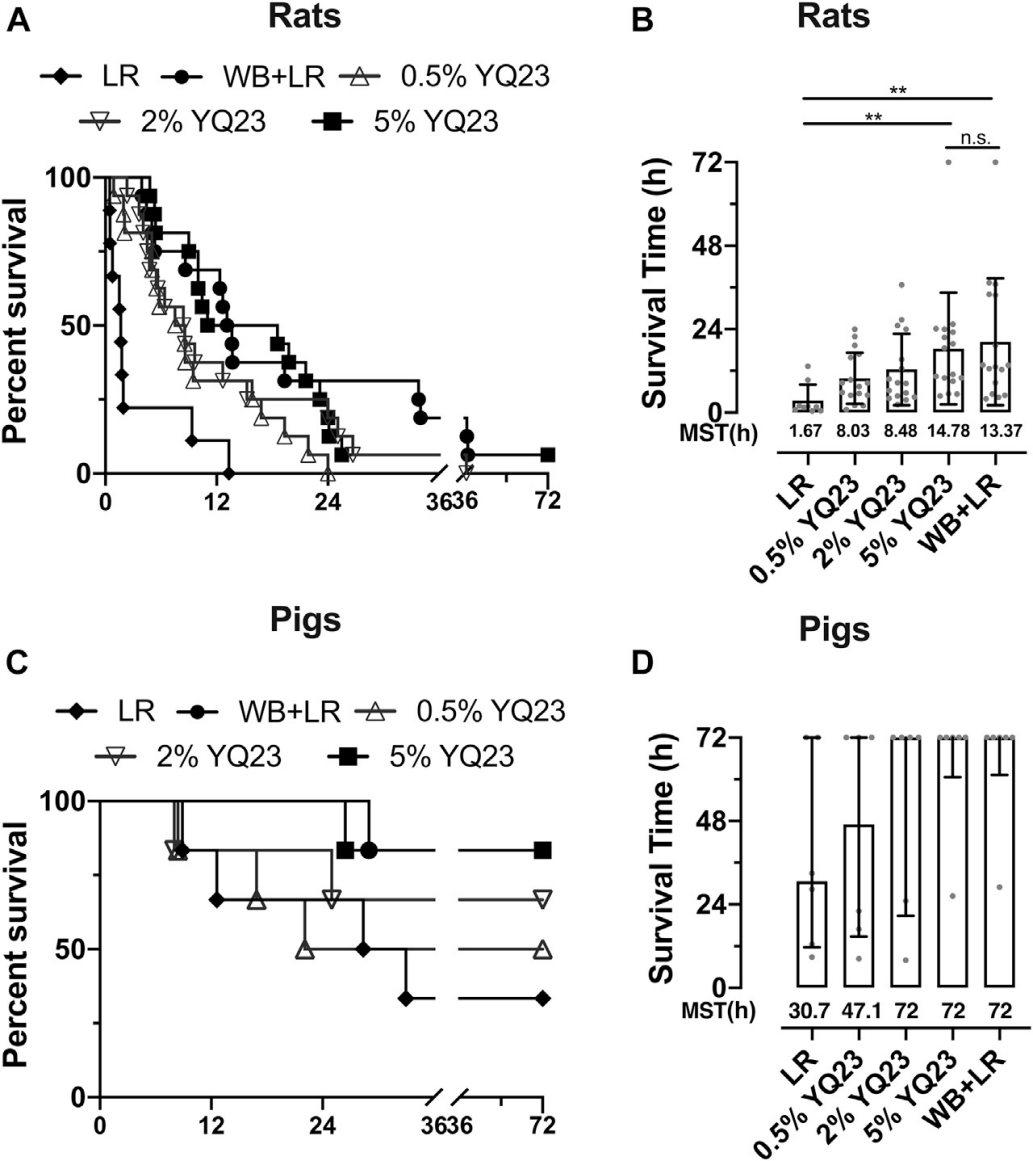
**YQ23 improved animal survival following uncontrolled hemorrhagic shock. (A)**, the survival curve of rats (*n* = 16/group); **(B)**, survival time of rats (*n* = 16/group); **(C)**, survival curve of pigs (*n* = 6/group); **(D)**, survival time of pigs (*n* = 6/group). LR, Lactate Ringer’s solution; WB, whole blood; MST, median survival time. *p < 0.05, **p < 0.01, n. s., not significant.

We observed the overall effects of YQ23 on UHS on survival to determine whether it could reduce early deaths. The results showed that YQ23 could prolong the survival time of rats compared with LR, there were 6/16 and 8/16 rats that survived over 12 h in 2 and 5% YQ23 group, respectively, and the median survival time was 8.5 and 14.8 h. WB + LR also significantly improved animal survival, and 11/16 rats survived more than 12 h, with a median survival time of 13.4 h; and effects of 5% YQ23 is equivalent to that of WB + LR (Figures 7A,B). These results indicate that YQ23 significantly reduces the early death of UHS rats and prolongs the survival time.

In UHS pigs, YQ23 tended to improve survival, too. Two pigs in the LR group survived less than 24 hours, with a median survival time of 30.69 hours. But in 5% YQ23 and WB + LR groups, all pigs survived more than 24 hours (Figures 7C,D).

### YQ23 Prolonged the Golden Hour for UHS Rescue

**FIGURE 8:**
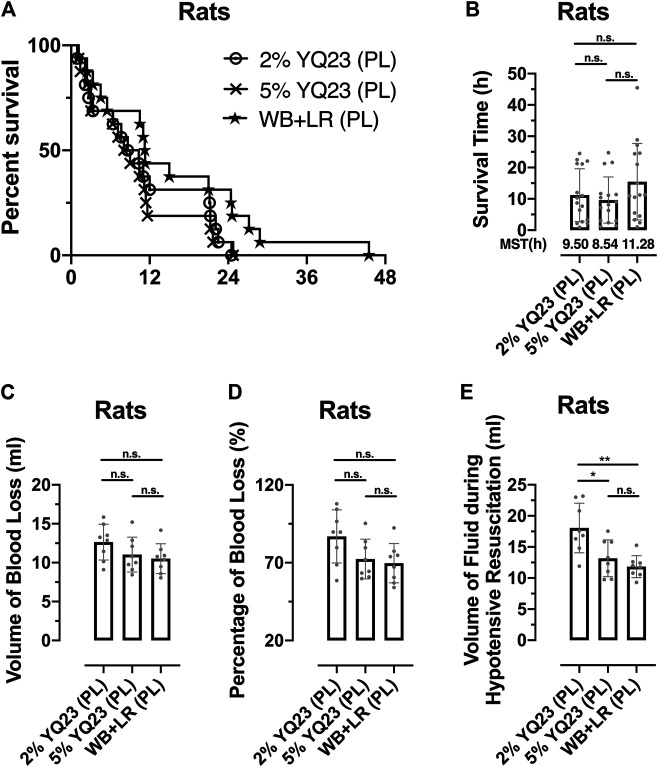
**YQ23 extended the Golden Hour for uncontrolled hemorrhagic shock. (A)**, survival curve of rats received prolonged hypotensive resuscitation (*n* = 16/group); **(B)**, the survival time of rats received prolonged hypotensive resuscitation (*n* = 16/group); **(C)**, the volume of blood loss in rats with prolonged hypotensive resuscitation (*n* = 8/group); **(D)**, percentage of blood loss in rats with prolonged hypotensive resuscitation (*n* = 8/group); **(E)**, volume of fluid demand in rats received prolonged hypotensive resuscitation (*n* = 8/group). LR, Lactate Ringer’s solution; WB, whole blood; MST, median survival time; PL, prolonged hypotensive resuscitation. *p < 0.05, **p < 0.01, n. s., not significant.

The timeframe is limited to 90 min using conventional fluid, which is not always enough for evacuation from the remote area or battlefield. In order to clarify whether YQ23 could help to extend the hypotensive resuscitation time window, we designed the Experiment Part 2 and the hypotensive resuscitation time was extended to 180 min (Figure 1). Consistent with the previous study, only 12.5% rats survived over 2 h for the hypotensive resuscitation using LR alone (data not shown), even though the infusion rate was increased to maintain MAP. Thus, we only adopted 2% YQ23, 5% YQ23, and WB + LR for further study. There were 75.0, 81.3, and 87.5% rats that survived after the 180 min-hypotensive resuscitation, and the median survival time was 9.50, 8.54, and 11.28 h, respectively, (Figures 8A,B). The total bleeding volume was increased due to the prolonged bleeding time, with 12.64 ± 2.30 ml, 11.03 ± 2.24, and 10.51 ± 1.92 ml in 2% YQ23, 5% YQ23 and WB + LR group (Figures 8C,D). However, the fluid demand in 5%YQ23 group and WB + LR group were equivalent (Figure 8E). These results show that by infusing YQ23 solution, the duration for pre-hospital treatment and hypotensive resuscitation can be extended.

## Discussion

Our previous studies have found that YQ23 is beneficial to hemorrhagic and septic shock (Li et al., 2017; Kuang et al., 2019). In order to observe whether YQ23 could benefit UHS before bleeding is controlled in the pre-hospital application, and clarify whether it could win opportunities for the subsequent treatment, this study replicated the rats and pigs UHS model and observed the effect of LR, WB + LR, and YQ23. It was found that compared with LR, YQ23 could effectively reduce bleeding, stabilize hemodynamics, and restore tissue oxygen utilization, improve organ function, and reduce early death. Thanks to its fine therapeutic effects, the hypotensive resuscitation duration could be extended to 180 min which helped to gain time for subsequent treatment.

Blood transfusion could improve the oxygen delivery, however, the tight blood source and the risks of infection limit its application (Hess et al., 2008). HBOC may play important roles in transfusion medicine and are beneficial to anemia, trauma, and hypoxia-related diseases (Jahr et al., 2021). YQ23 has a reduced affinity for oxygen (compared with free hemoglobin) and the p50 of hemoglobin in YQ23 is about 40 mmHg which is similar to RBC, and the oxygen releasing rate is faster than RBCs when oxygen partial pressure is low (Zhao et al., 2018), which suggested fine oxygen carrying capacity and facilitated rapidly restoring the oxygen content in hypoxic tissues. YQ23 increased the oxygen supply and restored tissue hypoxia, and won opportunity for subsequent treatment (Figure 4).

In addition to the oxygen-carrying function, we also found that YQ23 could reduce bleeding following UHS before the bleeding is not completely controlled, and the effects were comparable to WB + LR. There may be two reasons: firstly, limited fluid administration resulted in reduced risks of coagulopathy. Previous studies showed hemodilution effects of HBOC on coagulopathy, which is equivalent to LR and HEX (Moallempour et al., 2009; Morton et al., 2017; Meledeo et al., 2019). High dosages of HBOC could significantly increase the prothrombin time and activated partial thromboplastin time (Meledeo et al., 2019). In this study, the volume of YQ23 and WB + LR infused is smaller than LR, resulted in lower risks of hemodilution and coagulopathy (Figure 2). The content of methemoglobin in its storage solution is less than 4.8%, which is also related to reduced risks of coagulopathy (Moallempour et al., 2009). Besides, we found that MAP in YQ23 and WB + LR group was more stable (Figure 3), which helped to stabilize the microthrombus (Chatrath et al., 2015). Secondly, YQ23 might help to constrict the blood vessels. The prolonged shock will cause vascular hyporeactivity, dilated blood vessels contributes to further bleeding. Appropriate application of vasoconstrictor is helpful to reduce bleeding (Fangio et al., 2005; Gupta et al., 2017). In previous studies on controlled hemorrhagic shock and sepsis, YQ23 could restore the systemic vascular resistance index (SVRI), indicating that YQ23 may help to restore vascular reactivity (Li et al., 2017; Kuang et al., 2019).

YQ23 can significantly improve cardiac function, and stabilize hemodynamics. MAP in YQ23 group is stable, indicating steady blood perfusion in the liver, kidney, brain, and other important organs (Figures 4, 5). Previous studies have suggested that the duration of hypotensive resuscitation should be limited to 90 minutes in order to avoid further damage caused by continuous hypoperfusion and hypoxia (Li et al., 2011). Fortunately, HBOC could increase oxygen delivery, reduce blood loss while maintaining reasonable low blood pressure with low infusing volume, all these contribute to extending the Golden Hour for trauma patients.

Resuscitation using crystal fluid alone increases the risks of vascular leakage and tissue edema (Krausz et al., 2000). Clinical studies have combined crystal fluid and colloidal fluid in infusion, such as hypertonic saline plus dextran, HES plus LR, to reduce bleeding and fluid demand, and avoid tissue edema (Watters et al., 2006; Li et al., 2012). The colloidal property of hemoglobin solution during resuscitation might help to keep the liquid in the blood and reduce vascular leakage. Besides, study has shown that YQ23 reduces vascular leakage in hemorrhagic shock rats by protecting the endothelium (Zhao et al., 2020). The above factors may help to decrease fluid demand and maintain blood pressure, which is essential to maximize the efficiency of the limited liquid in pre-hospital conditions.

The side effects of HBOC have attracted researchers' attention. The severe adverse effect and long-term mortality was greater in previous HBOC-201 and PolyHeme trials, compared to conventional therapy (Dubé et al., 2019). Besides, hemoglobin quickly captures nitric oxide in the blood, and causes violent contraction of blood vessels and sudden rise in blood pressure, such as HemAssist (Schubert et al., 2002; Natanson et al., 2008). After decades of research and development, some HBOCs can be used for human under limited conditions (Freilich et al., 2009). In this study, MAP elevated smoothly and was stabilized during the slow infusion of YQ23. Besides, excessive free hemoglobin dimers and methemoglobin would increase the burden on the kidney and liver, which were responsible for clearance of the hemoglobin. The dimeric hemoglobin and methemoglobin in YQ23 was low and results also showed that there was no significant kidney injury and liver.

Some limitations in this experiment should be addressed: the effects of YQ23 during hypotensive resuscitation on the coagulopathy and hemostasis are unknown; the changes of some indicators in the late stages or before death have not been revealed because the variables were collected only within 2 h after treatment; the sample size of pig models was 6/group, and the statistical significance of some indicators is not clear; the effects of prolonged administration on physical status in were not observed Experiment Part 2. Besides, the YQ23 is a colloidal, whether the colloidal property of YQ23 could benefit UHS needs further investigation.

In summary, infusion of YQ23 benefited uncontrolled hemorrhagic shock and prevented early death in rats and pigs through increasing systemic oxygen supply, restoring oxygen metabolism, stabilizing hemodynamics, preventing organ injury and reducing bleeding. Besides, YQ23 helped to prolong the hypotensive duration and gain more time for subsequent treatment. Our study suggests that HBOC could be applied in early fluid resuscitation for trauma and shock.

## Data Availability

The original contributions presented in the study are included in the article/Supplementary Material, further inquiries can be directed to the corresponding authors.

## Fundings

This study is supported by the Key Program of National Natural Science Foundation of China (81730059), Key Project of the Army, PLA (BLJ19J008), Innovation Ability Training Program of the Army Medical Center (2019CSJSA0004).
